# Smart and smarter: improving on a classic egg shape model

**DOI:** 10.1007/s12064-025-00447-6

**Published:** 2025-09-26

**Authors:** Valeriy G. Narushin, Natalia A. Volkova, Alan Yu. Dzhagaev, Darren K. Griffin, Michael N. Romanov, Natalia A. Zinovieva

**Affiliations:** 1 Independent researcher, Zaporizhya, Ukraine; 2 L. K. Ernst Federal Research Center for Animal Husbandry, Dubrovitsy, Podolsk, Moscow Oblast, Russia; 3 School of Natural Sciences, University of Kent, Canterbury, Kent UK; 4 Animal Genomics and Bioresource Research Unit (AGB Research Unit), Faculty of Science, Kasetsart University, Chatuchak, Bangkok, Thailand

**Keywords:** Avian eggs, Egg geometry, Smart model, Standard egg shape, Main axiom of the mathematical formula of the bird’s egg

## Abstract

Smart’s model (SM) describing the geometry of avian eggs is, uniquely, based on physiological characteristics of eggs formation in oviduct walls transforming a sphere to an ellipsoid, to an ovoid. The purpose of this study was to revisit and perform a more in-depth examination of SM, providing a possible improvement in terms of reducing the number of initial parameters and compliance with geometric principles fundamental for bodies of revolution. SM requires measuring five egg parameters: length (*L*), maximum breadth (*B*), displacement of the central axis to the level of maximum breadth (*w*), and two radii of the egg at a point shifted by ¼L from the pointed (*r*) and blunt (*R*) ends, respectively. A practical test for the reproduction degree of three egg shape varieties using five-parameter model confirmed its maximum accuracy compared to all others. Modifications using four parameters (*L*, *B*, *w* and *r* or *B*_0_, which is egg diameter at ½*L*) were also relatively accurate, and only slightly inferior. Using three parameters (*L*, *B* and *w*) was clearly insufficient; however, one of our three-parameter models met the requirements of the “*Main Axiom of the mathematical formula of the bird’s egg*”. In our opinion, two of Smart’s postulates, the point of applying an oviduct force to provide the appropriate egg shape and the equality of *L* and the length of original ellipsoid, were used as fixed initial premises, which allowed to exclude many other possible options and to derive a mathematical model. Such an assumption arose according to the theoretical studies presented herein. Nevertheless, Smart’s formula derivation based on physiology of egg formation is a pioneering approach to the development of egg-shape models.

## Introduction

**Fig. 1:**
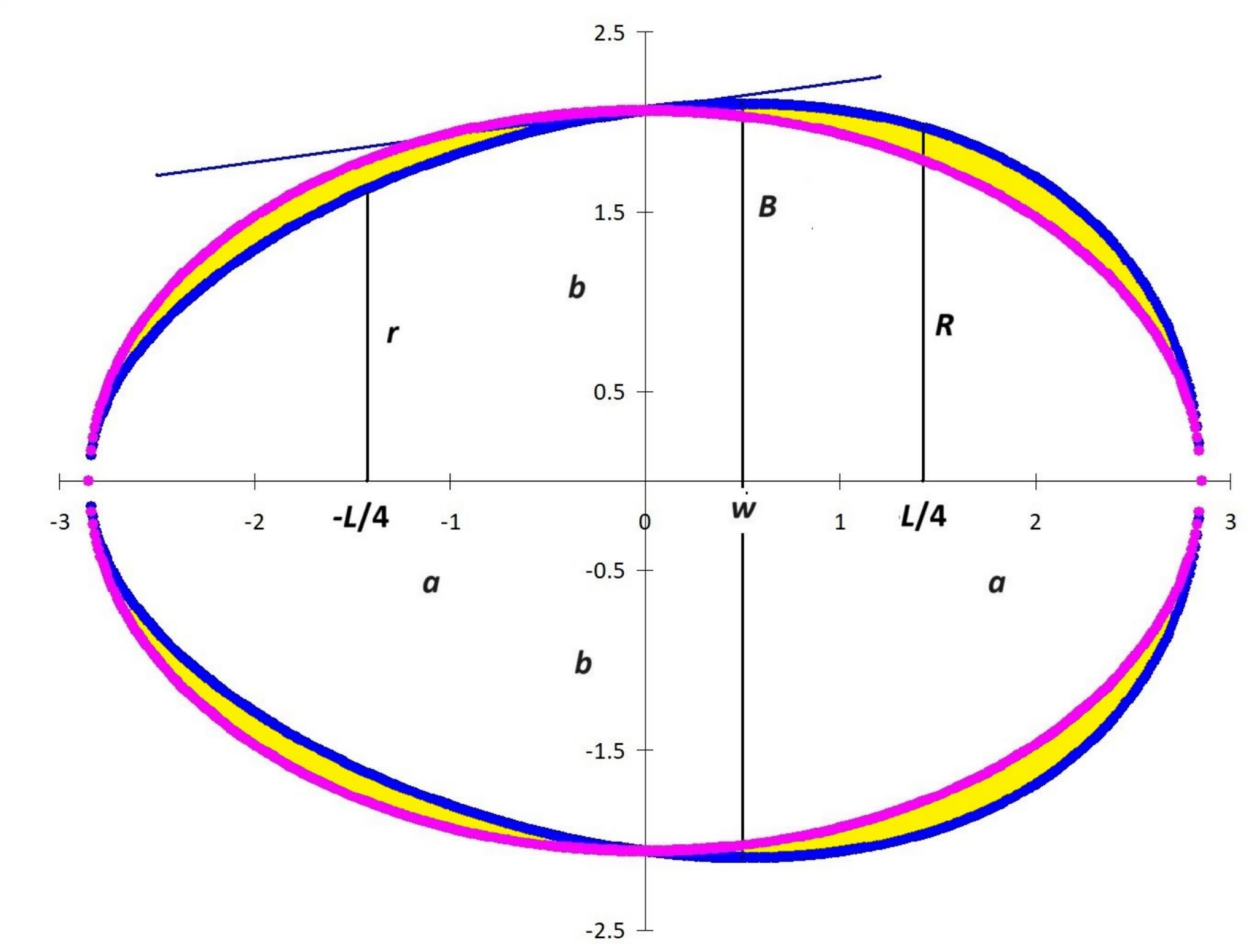
Schematic transformation of the egg contours (blue line) from an ellipse (purple line) according to the smart model

Iain Hugh Murray Smart (2 May 1928–24 December 2016) was a renowned Scottish physiologist, mountaineer and Arctic scientist (In Memoriam 2017). He published only three papers on the topic of mathematical description of the geometric profile of bird eggs (Smart 1969, 1991; Todd and Smart 1984) and derived his own egg shape model, hereinafter referred to as the Smart model (SM). When developing this model, smart considered the effect of the muscular structures of the oviduct in forming a certain shape to an egg before the glands of the mother’s body have yet to form the shell. According to Smart (1969, 1991), the initial shape of the egg is spherical, which, as a result of the primary axial action of the oviduct walls, transforms the sphere into an ellipsoid. After this, other oviduct walls are involved, transforming the ellipsoid to an egg-shaped (ovoid) form. When considering this mechanism of shape transformation, Smart (1969) made a number of assumptions:

1. When examining the longitudinal section of an egg in two dimensions, the area of this section remains unchanged, regardless of the shape being formed. This is due to the fact that the formation process begins with an already fully formed egg, albeit one not yet covered with a shell. In this regard, no matter how its shape changes, the area of the section remains unchanged. In a later work, Todd and Smart (1984) began to consider three-dimensional images of transformed figures, which is more logical and convenient for further mathematical analysis. Thus, we will further use the first postulate of the SM: the egg volume (*V*), regardless of the course of the formation process, remains unchanged. The following two, however, require revisiting.
2. The next assumption is related to the stage of transformation of the ellipsoid into an ovoid. According to Smart (1969), the egg length and its breadth in the central part remain unchanged. That is, the compressed part of the volume (the pointed end of the egg) “flows” into the blunt end (yellow zones on both sides of the vertical axis in Fig. 1), thereby facilitating its expansion, but neither the egg length, nor the size of the central diameter, suffers from such “flowing”.
3. Further compression of the ellipse continues at its midpoint, but at a certain angle, which forms the pointed part of the egg. As a result, smart included in his model the tangent of the angle of the tangential line to the midpoint on the surface of the egg, which also corresponds to the midpoint of the ellipse (Fig. 1). That is, this tangential line works as a lever that performs an oscillatory motion and relies on this point.

It should be noted that later works in this direction (e.g., Mao et al. 2006; Birkhead 2016; Deeming 2024) took the physiological stages of giving the egg its shape in the bird’s oviduct adopted by Smart as prerequisites for deriving a mathematical model of its contours. Given that we have no objections or alternatives to assumption (1) for the reasons provided, then the two remaining (2 and 3) require some additional research and confirmation. The adequacy of both the SM and the entire, so-called physiological approach to the mathematical description of the geometry of bird eggs will directly depend on this. Why we have questions about the validity of the assumptions (2) and (3) described above is easiest to explain in the form of the logical reasoning outlined below.

In his description of the physiology of the egg formation process, Smart (1969) pointed to the initial transformation of a sphere into an ellipsoid, which results in its decreased width and, accordingly, its increased length. For some reason, however, during the next transformation of the ellipsoid directly into an egg, the length remains unchanged, although the maximum diameter increases somewhat in size. Such a “frozen” state of the egg’s length seems somewhat illogical, given that quite recently, during the first stage of egg formation, it was actively increasing. It is reasonable to assume that both the egg length and width undergo slight changes during transit through the oviduct, eventually stabilizing at fixed values. However, based on the given schematic images of the further formation of the egg shape and, most importantly, following from the mathematical transformations undertaken by Smart (1969), it becomes obvious that the characteristic geometric dimensions, namely, the length and breadth at the central point, remain unchanged during the transformation of the ellipsoid into an egg. The modification of the shape is carried out due to the corresponding increase in the parameter of the maximum egg breadth. That is, the ellipsoid is as if inflated in breadth, leaving unchanged the parameters of the egg length and breadth at its central point that have already formed by this moment.

The issue of selecting and/or determining the angle included in the basic equation of the SM also remains insufficiently worked out. The final mathematical dependence was derived by Smart based on the proportion of certain sizes at a certain point lying on the surface of the egg. In this regard, the issue of choosing the application point of such forces remains open. Smart (1969) proposed a mathematical calculation of the magnitude of this angle by measuring two diameters of the egg, equidistant from its pointed and blunt ends. The adequacy of such a calculation remained unsupported by specific examples, however. In their later work, Todd and Smart (1984) carried out a practical check of their mathematical calculations on eggs of different shapes, but for such a description they used the Preston model (Preston 1953), transforming it from a parametric form to a Cartesian one. At the same time, Todd and Smart (1984) pointed out the similarity of their model with the approach of Preston (1953), due to the use of the mathematical product of the ellipse formula, plus some additional function that, in Preston (1953), has three varieties depending on the shape of the egg, i.e., polynomials of the 1st, 2nd and 3rd degrees. Weng et al. (2022) theoretically confirmed this similarity by performing the conversion process of the auxiliary function of the SM, including the angle of inclination of the tangential line to the point of pressure on the surface of the egg, by power series.

It should also be noted that, despite the sufficient popularity of the SM, it has remained remarkably under-explored. For example, there is practically no assessment of its application to describe the profiles of specific eggs. Of no small importance is the analysis of compliance with the basic mathematical and geometric principles. In our previous studies (Narushin et al. 2021), we proposed a basic concept of standardizing the egg model in the direction of developing a certain mathematical formula describing a standard geometric figure resembling the profile of a specific egg. Herewith, the main attention was paid to compliance with the basic geometric principles inherent in all standard geometric solids of revolution. In particular, a basic postulate was proposed that we (Narushin et al. 2023a) called the *“Main Axiom of the mathematical formula of the bird’s egg”* (hereafter the “Main Axiom”). This axiom essentially states that the extremum of the function describing the contour geometry of the egg should be equal to the half of its maximum breadth (*B*/2). Failure to comply with the Main Axiom principles leads to a violation of the basic egg dimensions in the model, most often, the egg maximum breadth (*B*) and, as a consequence, the derived calculated parameters, e.g., egg’s *V* and surface area (*S*). The principle underlying the concept of the Main Axiom has a purely geometric context and implies that the dimensions measured on an actual egg will fully conform to the same dimensions on its graphic contour. Particular attention is paid to the overall egg dimensions that, due to the symmetry of the egg relative to its longitudinal axis, include its length (*L*) and the maximum breadth (*B*). After all, it is the overall dimensions of geometric bodies that form a fundamental concept that determines their shape, volume, interaction with other objects, as well as applicability in various fields. These dimensions allow classifying bodies and predicting their behavior in various conditions. They determine whether an object will fit into a certain space, how it will interact with other objects, and how it will function as a whole. In mathematics and physics, overall dimensions are used for calculations, modeling and analysis of various phenomena. These dimensions are important for calculating packaging, transportation, storage and functional use of an object. However, quite often, when using different models describing the egg contours, the value of *B* is distorted (e.g., Narushin et al. 2020b). In this respect, at the stage of development, testing and analysis of the corresponding mathematical model, one simple mathematical property should be verified: does the location and maximum size of the function describing the egg contours coincide with the actual *B* value involved in these calculations. This operation is quite simple to carry out mathematically by finding the derivative and equating it to 0. This is precisely the principle of the Main Axiom.

The aim of the current study was thus a theoretical analysis of the SM for its compliance with mathematical and geometric principles. We also aimed to provide an experimental verification of the possibility of using it for the practical description of actual contours inherent in bird eggs of various shapes.

## Theory

### SM and its parameters analysis

According to Smart (1969), the mathematical formula describing the geometry of a bird’s egg is as follows:

1 $$\frac{{x^{2} }}{{a^{2} }} + \frac{{y^{2} }}{{(b + x\tan \theta )^{2} }} = 1$$

wherefrom

2 $$y = \pm \frac{b + x\tan \theta }{a}\sqrt {a^{2} - x^{2} }$$

where *a* and *b* represent lengths of the ellipse’s semi-major and semi-minor axes, respectively; and *θ* is the angle of inclination of the tangential line to the horizontal at the midpoint on the surface of the formed egg (Fig. 1).

In view of the development of some standard in the designation of geometric parameters of bird eggs (e.g., Narushin et al. 2021, 2023a), we decided to transform Eq. (2) into a more unified form based on the conditions that *L* = 2*a* and *B*_0_ = 2*b*, as follows:

3 $$y = \pm \frac{{B_{0} + 2x\tan \theta }}{2L}\sqrt {L^{2} - 4x^{2} }$$

At the same time, it should be noted that the median diameter (*B*_0_) of the egg at point *x* = 0 is quite inconvenient in terms of measuring it. It is advisable and more practical to replace it with *B* that corresponds to point *x* = *w* for subsequent mathematical transformations. Thus, *w* is the distance by which *B* is shifted from its vertical axis (Fig. 1). Substituting these conditions, i.e., *x* = *w* and *y* = *B*/2, into Eq. 3, we obtain:

4 $$B_{0} = \frac{BL}{{\sqrt {L^{2} - 4w^{2} } }} - 2w\tan \theta$$

Considering the obtained Eq. 4, SM (Eq. 3) can be transformed into the following:

5 $$y = \pm \left( {\frac{B}{{2\sqrt {L^{2} - 4w^{2} } }} + \frac{\tan \theta (x - w)}{L}} \right)\sqrt {L^{2} - 4x^{2} }$$

Among other parameters of a slightly modified SM (Eq. 5), which can be relatively easily measured and/or calculated (Narushin et al. 2020b, 2021, 2023a), there is the most “mysterious” component, i.e., tan*θ*. The accuracy of contour reproduction depends on the correctness of its definition or selection. Smart (1969) proposed the algorithm for its calculation by additionally measuring two radii of the egg spaced by the same distance from the pointed and blunt ends. Let us consider the validity of this calculation in more detail. As additionally measured parameters, we select the radii *r* and *R* spaced from the central vertical axis of the egg at distances *x* = –*L*/4 and *x* = *L*/4, respectively (Fig. 1). In a number of our previous works (Narushin et al. 2020b, 2021, 2023a), we demonstrated the high informational significance of these parameters. Then, according to the formulas proposed by Smart (1969) and the parameters chosen in our example, the value of tan*θ* can be expressed by the following formula:

6 $$\tan \theta = \frac{4}{\sqrt 3 } \cdot \frac{R - r}{L}$$

As a result, Eq. 5 will look like:

7 $$y = \pm \left( {\frac{B}{{2\sqrt {L^{2} - 4w^{2} } }} + \frac{4(R - r)}{{\sqrt 3 L^{2} }}(x - w)} \right)\sqrt {L^{2} - 4x^{2} }$$

In order not to confuse this representation of SM with the original formula (Eq. 5), we decided to denote Eq. (7) SM^*r*^.

In addition to the considered version of the definition of tan*θ* proposed by Smart (1969), the value of the tangent of the inclination angle of the tangential line (*θ*) at the point (0; *B*_0_/2) can be determined from Eq. (5) by substituting the corresponding values *x* and *y*:

8 $$\tan \theta = \frac{{BL - B_{0} \sqrt {L^{2} - 4w^{2} } }}{{2w\sqrt {L^{2} - 4w^{2} } }}$$

Substituting the obtained value of tan*θ* into SM (Eq. 5), we will get:

9 $$y = \pm \frac{1}{L}\left( {\frac{{BL - B_{0} \sqrt {L^{2} - 4w^{2} } }}{{2w\sqrt {L^{2} - 4w^{2} } }}x + \frac{{B_{0} }}{2}} \right)\sqrt {L^{2} - 4x^{2} }$$

Again, in order not to confuse this modification of the model with the previous ones, we will designate it (Eq. 9) as $\text{SM}^{B_0}$. To use this version of the model, in addition to the parameters *L*, *B* and *w*, the diameter of the egg at its midpoint (*B*_0_) should also be measured, which is quite problematic. In this regard, we decided to apply a few approaches due to which the value of *B*_0_ could be excluded from the set of the calculated parameters.

By means of simple transformations, we can write Eq. 9 in the following form that will significantly facilitate its subsequent analysis:

10 $$\frac{y}{B} = \pm \left( {\left[ {\frac{1}{{\sqrt {1 - 4\left( \frac{w}{L} \right)^{2} } }} - \frac{{B_{0} }}{B}} \right] \cdot \frac{1}{{2\frac{w}{L}}} \cdot \frac{x}{L} + \frac{1}{2} \cdot \frac{{B_{0} }}{B}} \right)\sqrt {1 - 4\left( \frac{x}{L} \right)^{2} }$$

For a full analysis of the obtained Eq. 10, we need to determine the possible variation of the *w*/*L* and *B*_0_/*B* ratios. As for the former index, the boundaries of its variability have been studied by us quite extensively (Narushin et al. 2021, 2022, 2023a, b), as a result of which it can be stated that the possible variation of *w*/*L* can be accepted at the level of [0 … 0.2]. In the case of the *B*_0_/*B* ratio, its possible variation can be determined based on the conditional boundary shapes of the geometric profiles of bird eggs. We have demonstrated theoretically (Narushin et al. 2021, 2023a) and confirmed practically (Narushin et al. 2023b) that the actual profile of the pointed end among the entire variety of bird eggs is in the interval between two geometric figures: (1) a parabola, and (2) an ovoid determined by the Hügelschäffer formula (Narushin et al. 2020b). Since, for both formulae, we are interested in the condition *x* = 0, *y* = *B*_0_/2, we will have:

- for the equation of a parabola, that is, the minimum permissible values of (*B*_0_/*B*)_min_,

11 $$y = \pm \frac{B}{2} \cdot \sqrt {\frac{L - 2x}{{L + 2w}}}$$

and after appropriate transformations, leaving only the upper part of the profile,

12 $$\left( {\frac{{B_{0} }}{B}} \right)_{\min } = \frac{1}{{\sqrt {1 + 2\frac{w}{L}} }}$$

- for the Hügelschäffer model, that is, the minimum permissible values of (*B*_0_/*B*)_max_,

13 $$y = \pm \frac{B}{2}\sqrt {\frac{{L^{2} - 4x^{2} }}{{L^{2} + 8wx + 4w^{2} }}}$$

and its analogous transformation will give

14 $$\left( {\frac{{B_{0} }}{B}} \right)_{\max } = \frac{1}{{\sqrt {1 + 4\left( \frac{w}{L} \right)^{2} } }}$$

An intermediate variant was added to the obtained boundary intervals of variation (Eqs. 12 and 14) for average values of (*B*_0_/*B*)_av_:

15 $$\left( {\frac{{B_{0} }}{B}} \right)_{av} = \frac{1}{2}\left( {\frac{1}{{\sqrt {1 + 2\frac{w}{L}} }} + \frac{1}{{\sqrt {1 + 4\left( \frac{w}{L} \right)^{2} } }}} \right)$$

**Fig. 2:**
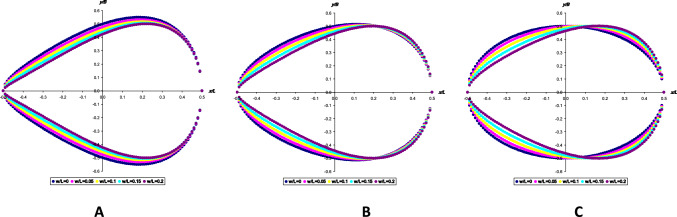
Theoretical egg profiles constructed according to the smart model *B*_0_ expressed as Eq. 10 for all possible values of *w*/*L* with the *B*_0_/*B* parameter ratio as calculated according to Eq. 12 (**A**), Eq. 15 (**B**) and Eq. 14 (**C**)

By dividing the *w*/*L* variation interval into five equal intervals with a step of 0.05 and substituting each of the values into three conditional geometric egg profiles (Eqs. 12, 14 and 15), we formed a database of possible parameter variations, according to which theoretical egg profiles were constructed (Fig. 2) that were described by $\text{SM}^{B_0}$ expressed as Eq. 10.

The analysis of the obtained theoretical egg profiles demonstrates the greatest visual similarity with actual bird eggs (Fig. 2C), when the dependence of *B*_0_/*B* on *w*/*L* is expressed by the Hügelschäffer model (Eq. 14). The disadvantage of all three graphical representations of egg contours is the fact that *B* goes beyond the possible limits of its variability, which, for the representation of $\text{SM}^{B_0}$ in the form of Eq. 10, should be within *y*/*B* = − 0.5 … 0.5. Otherwise, it turns out that the obtained values of *y* are higher than the actual values ± *B*/2 and are shifted away from their correct location at the point *x* = *w*. That is, in the graphical dependence there is a violation of the dimensions of an actual egg. This indicates a violation of the Main Axiom, which should be verified by performing a separate, more thorough analysis.

However, before proceeding to the analysis of the extremum of the function (Eq. 10), we decided to conduct a similar visualization of SM, when the tangent of the angle *θ* is determined by two additional measurements of the egg radii (Eq. 6). For this, we will consider SM^*r*^ expressed by Eq. (7), which after some mathematical transformations will take the following form:

16 $$\frac{y}{B} = \pm \left( {\frac{1}{{2\sqrt {1 - 4\left( \frac{w}{L} \right)^{2} } }} + \frac{4}{\sqrt 3 }\left( {\frac{R}{B} - \frac{r}{B}} \right)\left( {\frac{x}{L} - \frac{w}{L}} \right)} \right)\sqrt {1 - 4\left( \frac{x}{L} \right)^{2} }$$

Keeping the variation interval of *w*/*L* similar to the previous example, it remains for us to determine the boundaries of possible changes in the *R*/*B* and *r*/*B* ratios. Again, returning to the geometric models of bird egg contours we considered with their possible variations in the shapes of the pointed end from a parabola to a Hügelschäffer ovoid (Narushin et al. 2021, 2023a, b), the boundary values of *r*/*B* can be obtained using these geometric figures. The only thing that should be taken into account is the opposite arrangement of the egg contours. Use of SM assumes the location of the pointed end in the sector of negative *x* values, while using Eqs. 11 and 13 will place the blunt part of the egg in this sector. That is, when calculating, we should take this fact into account, accepting the values *x* = *L*/4 and *y* = *r* for the pointed part and *x* = − *L*/4 and *y* = *R* for the blunt part. In contrast to the pointed part, the blunt part for all the variety of shapes corresponds to the Hügelschäffer ovoid (Narushin et al. 2021, 2023a, b) and can be calculated according to Eq. 13 for any variations in the shape of the acute end of the bird’s egg. Then, for the pointed end we have:

- for the equation of a parabola (Eq. 11) at *x* = *L*/4 and *y* = *r*

17 $$\frac{r}{B} = \frac{1}{{2\sqrt {2\left( {1 + 2\frac{w}{L}} \right)} }}$$

- for the Hügelschäffer model (Eq. 13) at *x* = *L*/4 and *y* = *r*

18 $$\frac{r}{B} = \frac{1}{4} \cdot \sqrt {\frac{3}{{1 + 2\frac{w}{L} + 4\left( \frac{w}{L} \right)^{2} }}}$$

Similar to the previous example, let us also take for analysis an intermediate variant expressed as the arithmetic mean of Eqs. 17 and 18:

19 $$\frac{r}{B} = \frac{1}{4}\left[ {\frac{1}{{\sqrt {2\left( {1 + 2\frac{w}{L}} \right)} }} + \frac{1}{2} \cdot \sqrt {\frac{3}{{1 + 2\frac{w}{L} + 4\left( \frac{w}{L} \right)^{2} }}} } \right]$$

The value of the radius *R* at the blunt end of the egg will be the same for all shape varieties of the pointed end and will be determined by substituting into Eq. 13 the values *x* = − *L*/4 and *y* = *R*:

20 $$\frac{R}{B} = \frac{1}{4} \cdot \sqrt {\frac{3}{{1 - 2\frac{w}{L} + 4\left( \frac{w}{L} \right)^{2} }}}$$

**Fig. 3:**
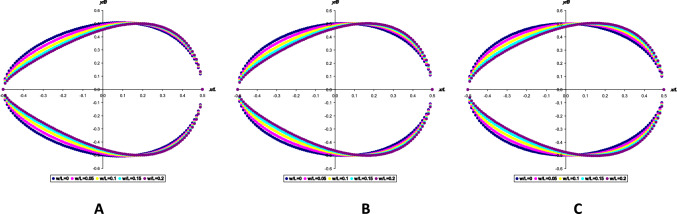
Theoretical egg profiles constructed according to the SM^*r*^ expressed as Eq. 16 for all possible values of *w*/*L* with the parameter ratio *R*/*B* calculated according to Eq. 20 and *r*/*B* calculated according to Eq. 17 (**A**), Eq. 19 (**B**) and Eq. 18 (**C**)

By dividing the *w*/*L* variation interval into five equal parts with an increment of 0.05 and substituting each of the values into three conditional geometric egg profiles (Eqs. 17, 18 and 19) for *r*/*B* index and into Eq. 20 for the *R*/*B* index, we formed a database of possible parameter variations, according to which theoretical egg profiles were constructed (Fig. 3) as described by SM^*r*^ expressed by Eq. 16.

Similar to the previous variant (Fig. 2), Fig. 3 also shows a violation of *B* and a shift in the location of the *B* axis away from its correct location in the coordinate *x* = *w*, although all three considered variants (Fig. 3A–C) visually reflect the actual egg contours quite plausibly. A more detailed analysis of the numerical data, on the basis of which the graphic contours were constructed similar to those shown in Fig. 2, demonstrated a more accurate reproduction when using the Hügelschäffer model (Eq. 18) to calculate the value of *r*/*B*.

Considering the fact that the parameter *B*_0_/*B* in Eq. 10, as well as the parameters *r*/*B* and *R*/*B* in Eq. 16, should be expressed based on the Hügelschäffer model (respectively Eqs. 14, 18 and 20), the variations of $\text{SM}^{B_0}$ and SM^*r*^ can be represented as two formulae below, each of which includes three basic egg parameters: *L*, *B* and *w*. To simplify identification, we will assign them the appropriate indices that will allow us to identify the original modification of the model:

- $$\text{SM}^{B_{02}}$$

21 $$y = \pm \frac{B}{{2\sqrt {L^{2} + 4w^{2} } }}\left( {\frac{{\sqrt {L^{2} + 4w^{2} } - \sqrt {L^{2} - 4w^{2} } }}{{w\sqrt {L^{2} - 4w^{2} } }}x + 1} \right)\sqrt {L^{2} - 4x^{2} }$$

- $$\text{SM}^{r_2}$$

22 $$y = \pm B\left( {\frac{1}{{2\sqrt {L^{2} - 4w^{2} } }} + \left[ {\frac{1}{{\sqrt {L^{2} - 2wL + 4w^{2} } }} - \frac{1}{{\sqrt {L^{2} + 2wL + 4w^{2} } }}} \right]\frac{x - w}{L}} \right)\sqrt {L^{2} - 4x^{2} }$$

We decided to evaluate the accuracy of reproduction of actual egg contours using each of the above equations (i.e., Eqs. 21 and 22) by conducting the experimental studies outlined below.

### SM and the main axiom

We consistently return to the issue of the universality criterion of the geometric model of bird eggs based on the fact that only a combination of two indicators, i.e., (1) the accuracy of contour reproduction and (2) compliance with the principles of mathematical laws for geometric figures, will allow us to classify this or that dependence as universal. At the same time, none of these components, showing positive results separately from each other, can outweigh the significance of compliance with both criteria in combination. In this regard, the issue of analytical verification of the SM for compliance with the Main Axiom, in other words, with the correctness of the location and adequacy of *B*, was given the closest attention.

For our analysis, the basic equation of SM was chosen, including the parameters *B* and *w* (Eq. 5), since they carry the main information component in the further analysis. The derivative of this function (*y’*) will have the following form:

23 $$y^{\prime} = \frac{\tan \theta }{L}\sqrt {L^{2} - 4x^{2} } - \left( {\frac{B}{{2\sqrt {L^{2} - 4w^{2} } }} + \frac{\tan \theta (x - w)}{L}} \right)\frac{4x}{{\sqrt {L^{2} - 4x^{2} } }}$$

According to the Main Axiom conditions, the extremum point (*y’* = 0) must correspond to the value *x* = *w*. Then, Eq. 23 can be rewritten as follows:

24 $$\frac{\tan \theta }{L}\sqrt {L^{2} - 4w^{2} } - \frac{2Bw}{{L^{2} - 4w^{2} }} = 0$$

After mathematical transformations of this equation (Eq. 24), the Main Axiom conditions will be fulfilled when

25 $$\tan \theta = \frac{2BLw}{{(L^{2} - 4w^{2} )\sqrt {L^{2} - 4w^{2} } }}$$

Substituting the obtained dependence (Eq. 25) into Eq. 5, we obtain the equation of SM^MA^ that meets the Main Axiom principles:

26 $$y = \pm \frac{B}{2}\left( {1 + \frac{4w}{{L^{2} - 4w^{2} }}(x - w)} \right)\sqrt {\frac{{L^{2} - 4x^{2} }}{{L^{2} - 4w^{2} }}}$$

A practical test for the reproduction accuracy of actual egg contours using SM^MA^ (Eq. 26) was also planned to be carried out by conducting experimental studies.

### Does L egg remain constant while an egg is formed in a bird’s oviduct?

As stated above, Smart (1969) proceeded from the fact that *L* remains unchanged during the process of pressure of the oviduct walls and the formation of an ovoid profile by squeezing the breadth of the egg at the central point, along the *y*-axis. At the same time, *V* remains constant. That is, if we consider Fig. 1, the volume of the “yellow” zones lying to the left of the *y*-axis should correspond to the volume of the “yellow*"* zones lying to the right of the *y*-axis. Otherwise, the difference between half the volume of the ellipsoid (*V*_*el*_/2) and the volume of the pointed part of the egg (*V*_*p*_) should be equal to the difference between the volume of the blunt part of the egg (*V*_*b*_) and *V*_*el*_/2:

27 $$\frac{{V_{el} }}{2} - V_{p} = V_{b} - \frac{{V_{el} }}{2}$$

Considering that the sum of *V*_*p*_ and *V*_*b*_ is nothing other than *V* of the entire egg, it remains for us to prove that *V*_*el*_ and *V* expressed by SM will have the same value.

Taking into account the notations we have adopted, *V*_*el*_ will be written as:

28 $$V_{el} = \frac{1}{6}\pi LB_{0}^{2}$$

As for the ovoid described by SM, the calculation formula for its volume should be determined. For the convenience of performing a possible subsequent analysis, we will determine the calculation formulae for the volume of each of its parts (*V*_*p*_ and *V*_*b*_) separately. For this, we can apply the formulae of integral geometry, according to which and based on Fig. 1:

29 $$V_{p} = \pi \int\limits_{{ - \frac{L}{2}}}^{0} {y^{2} dx}$$

30 $$V_{b} = \pi \int\limits_{0}^{\frac{L}{2}} {y^{2} dx}$$

Using the original formula of SM (Eq. 3), we obtain:

31 $$V_{p} = \frac{\pi }{{4L^{2} }}\int\limits_{{ - \frac{L}{2}}}^{0} {(B_{0} + 2x\tan \theta )^{2} (L^{2} - 4x^{2} )dx}$$

32 $$V_{b} = \frac{\pi }{{4L^{2} }}\int\limits_{0}^{\frac{L}{2}} {(B_{0} + 2x\tan \theta )^{2} (L^{2} - 4x^{2} )dx}$$

The result of integrating Eqs. 31 and 32 will give the desired calculation formulae:

33 $$V_{p} = \frac{\pi L}{4}\left( {\frac{1}{3}B_{0}^{2} - \frac{1}{4}B_{0} L\tan \theta + \frac{1}{15}L^{2} \tan^{2} \theta } \right)$$

34 $$V_{b} = \frac{\pi L}{4}\left( {\frac{1}{3}B_{0}^{2} + \frac{1}{4}B_{0} L\tan \theta + \frac{1}{15}L^{2} \tan^{2} \theta } \right)$$

The sum of Eqs. 33 and 34 will correspond to *V* of the whole egg:

35 $$V = \frac{\pi L}{6}\left( {B_{0}^{2} + \frac{1}{5}L^{2} \tan^{2} \theta } \right)$$

Taking into account the mathematical transformations carried out, the condition of equality of the volumes of the “yellow” zones expressed as the equality of *V*_*el*_ and *V* (Eq. 27) will be written as:

36 $$\frac{1}{6}\pi LB_{0}^{2} = \frac{\pi L}{6}\left( {B_{0}^{2} + \frac{1}{5}L^{2} \tan^{2} \theta } \right)$$

The observance of this equality (Eq. 36) is possible only when $\tan \theta = 0$ and, respectively, $\theta = 0$, and the egg has the shape of an ellipsoid.

Thus, we have theoretically demonstrated the fallacy of the assumption that *L* remains unchanged after the formation of the ovoid from the ellipsoid. Let us check how the *L* will change, for which in the calculation formula for the volume of the ellipsoid (Eq. 28) instead of the parameter *L*, we will introduce the parameter *L*_*el*_ that will characterize the length of the ellipsoid. Then, equality (36) will be modified to the following equation:

37 $$\frac{1}{6}\pi L_{el} B_{0}^{2} = \frac{\pi L}{6}\left( {B_{0}^{2} + \frac{1}{5}L^{2} \tan^{2} \theta } \right)$$

wherefrom

38 $$L_{el} = L\left[ {1 + \frac{1}{5}\left( {\frac{L}{{B_{0} }}} \right)^{2} \tan^{2} \theta } \right]$$

Considering that the value in the Eq. 38 squared brackets will always be greater than 1, *L*_*el*_ will exceed *L*. Thus, if we follow Smart’s (1969) assumption about the equality of the volumes of the “yellow” zones (Fig. 1), the mother bird should also act on the egg along the horizontal axis, compressing it lengthwise.

Without a doubt, the process of giving the egg its final form is quite complex for a final interpretation. In this case, fixing some mathematical initial data is a completely justified and extremely logical operation for subsequent mathematical transformations and subsequent analysis. Nevertheless, the optimal approach for deriving a geometric model and related calculation formulae, in our opinion, may be measurements of the main parameters of an already laid egg that has a calcified shell.

In this case, we used the approach of comparing the volumes of the original ellipse and the already formed egg only as an auxiliary mechanism for answering the question of the constancy (or, conversely, variability) of the egg length in the process of giving it the final shape. However, given that the derivation of Eq. 35 was carried out using the classical method of integral calculation of volumes of solids of revolution, this formula is adequate for determining the volume of a bird’s egg, as well as for any geometric body whose contours conform to the Smart model. In this case, it should be taken into account that the values of the geometric dimensions will correspond to the solid of revolution whose volume we want to determine. That is, if we were talking about a shellless egg inside a bird’s oviduct, the *L* value corresponded to the length of this shellless object, and *B*_0_ to its breadth at the central point. Then, for a laid egg, the values of these quantities should be measured taking into account the thickness of the shell. If we generalize the use of Eq. 35 as a calculation formula for the volume of any solid of revolution whose contours are described by the Smart model, the values of *L* and *B*_0_ are the length and corresponding breadth of this particular solid.

Overall, our further objective was reduced to checking the adequacy, simplicity and accuracy of reproduction of the SM in its obtained modifications: (1) SM^*r*^ (Eq. 7); (2) $\text{SM}^{B_0}$ (Eq. 9); (3) $\text{SM}^{B_{02}}$ (Eq. 21); (4) $\text{SM}^{r_2}$ (Eq. 22); and (5) SM^MA^ (Eq. 26).

## Materials and methods

**Fig. 4:**
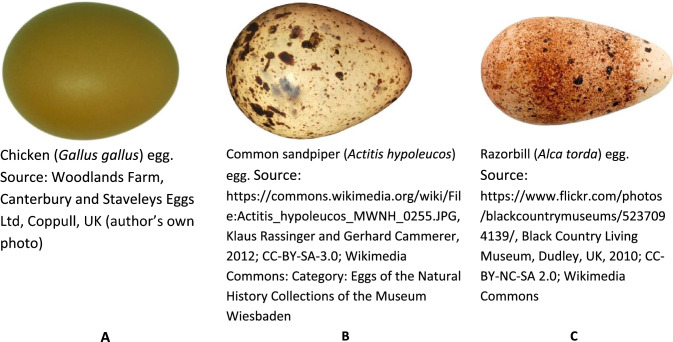
Three eggs of characteristic shapes used in the present experiment: ovoid (**A**), conical (**B**), and pear-shaped, or pyriform (**C**)

Our methodology is based on our previous work on digital profiles (Narushin et al. 2020a, b, 2021, 2022, 2023a, b). Some of these, e.g., chicken egg profiles, conform to the shape of a classic ovoid, others are more reminiscent of a pyriform (pear) shape, while others can be characterized as conical (a kind of “symbiosis” of the first and second versions). Since the images used for the analysis were taken from different studies, the methodological approaches to obtaining their digital images also differed. The image of a chicken egg (Fig. 4A) was obtained after photographing it with a digital camera and post-processing using MatLab. This allows to process the image and compute the geometric parameters by measuring the corresponding diameters in 1-pixel increments along its length (Narushin et al. 2020a). The RGB (Red, Green and Blue) images of the chicken eggs were firstly converted to grey-scale images for the edge detection purpose. The Sobel edge detection technique (Chandwadkar et al. 2013) was then applied on the grey-scale images to determine the outer contour of the egg over the images (Narushin et al. 2020a).

Images of the common sandpiper (Fig. 4B) and razorbill (Fig. 4C) eggs were scaled to a uniform length, *L* = 57 mm, which was 215 pixels. The corresponding egg diameters were measured along the egg length with the microsoft picture manager software in 1-pixel increments. The egg outlines in the images were obtained via manual tracing.

As a result, we decided to select a few characteristic shapes (Fig. 4) that would represent model objects when testing the practical use of the equations we selected, i.e., modifications of SM. The digital contours of the chicken egg (Fig. 4A), both the original and those created in accordance with various SM variations, were reduced to a single scale in the pixel measurement system. Since the *L* value of the chicken egg was 398 pixels, the assessment of the conformity of its contours with the corresponding SM modification was performed by the degree of conformity of the 398 points that comprised the contours of the real egg and its geometric display calculated using one of the following formulae: Eq. 7, Eq. 9, Eq. 21, Eq. 22 and Eq. 26. The geometric profiles of the common sandpiper (Fig. 4B) and razorbill (Fig. 4C) eggs were built in a similar manner, using 215 points on their contours corresponding to the results of the measurements of the images of actual eggs.

Since each of the eggs (respectively, Fig. 4A–C) was used to perform a separate comparative analysis of the correspondence of an actual contour to one of the theoretical modifications of Smart’s model, this approach was considered acceptable.

For each egg, its geometric shape was generated using each of the five SM modifications and compared with the actual digital profile. An approximate mean percentage error, *ε* (e.g., Makridakis et al. 1982), was used to evaluate how well each hypothesized egg profile matched the actual one as follows:

39 $$\varepsilon = \frac{1}{k} \cdot \sum\limits_{1}^{n} {\left| {\frac{{v_{1} - v_{2} }}{{v_{1} }}} \right|} \cdot 100\%$$

where *k* represents a number of *x* points on the horizontal axis, and *v*_1_ and *v*_2_ represent the pertinent values of *y* that were obtained, respectively, from (1) a direct measurement of the egg profile and (2) calculation using the appropriate SM modification. The *x*-values conformed to the points at which the original measurements of each image were taken. In particular, for the chicken egg (Fig. 4A), the comparison was made over 398 points, and for the common sandpiper (Fig. 4B) and razorbill (Fig. 4C), over 215 points.

## Results and discussion

The visualization results for geometric profiles obtained using various SM modifications are presented in Table 1 in comparison with actual contours of three types of bird eggs. Each profile was characterized by the respective value of *ε* (%).

**Table 1:**
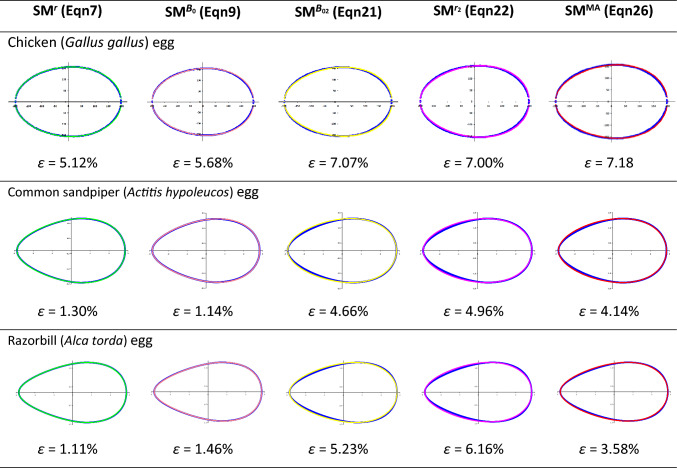
Results of visualization of geometric profiles obtained using various Smart model’s (SM) modifications (green, coral, yellow, purple and red lines, respectively), in comparison with the actual contours of three types of bird eggs (blue line)

The mathematical description results for the actual contours of three bird egg types demonstrated their most accurate reproduction using the classical SM^*r*^ representation (Eq. 7), for which five main parameters should be measured: *L*, *B*, *w*, *R* and *r*. Also, it was surprising that the pyriform eggs, more complex in terms of mathematical representation (Narushin et al. 2021), showed a smaller error than the usual ovoid of a chicken egg. That is, we can safely say that this model is suitable for defining eggs of any shape. The complexity of this model (Eq. 7), however, lies in the need to measure five parameters. Moreover, if *L* and *B* of the egg are easy to measure with conventional measuring instruments like a caliper, the remaining three parameters require a more involved approach. We recommend using a digital image of the egg with subsequent pixel-by-pixel measurement of the parameters of interest (Narushin et al. 2020a, b, 2021, 2022, 2023a, b, 2024a, b). This approach, nevertheless, may not always be applicable in practice. In this regard, one of the tasks we pursued was to reduce the number of initial measurements and/or replace inconvenient (complex) measurements with simpler ones. For example, to use $\text{SM}^{B_0}$ (Eq. 9), it was necessary to measure four parameters (*L*, *B*, *w*, and* B*_0_), and for the remaining ones (Eqs. 21, 22 and 26), three (*L*, *B* and* w*). While, for the four-parameter model (Eq. 9), the result turned out to be quite acceptable in terms of contour reproduction accuracy, for three-parameter ones (Eqs. 21, 22 and 26), the *ε* value was clearly too high.

Of greatest interest to us were the results of using SM^MA^ (Eq. 26), as it was the only one satisfying the Main Axiom requirements. Although of all three-parameter models (Eqs. 21, 22 and 26) SM^MA^ (Eq. 26) demonstrated the highest accuracy, the *ε* value was still clearly higher (sometimes more than three-fold) than when using SM^*r*^ (Eq. 7). Visually (Table 1), it is evident that the results of egg contour reproduction using SM^MA^ (Eq. 26) have maximum differences in the range of *r* values. In this regard, we decided to check how much the result would be improved, including in the matter of compliance with the Main Axiom conditions, if we somehow additionally introduce the parameter *r* into SM^MA^.

As a preliminary step, we decided to evaluate SM^*r*^ (Eq. 7) in terms of the ratio of its parameters for compliance with the Main Axiom requirements, similar to what we performed when deriving SM^MA^ (Eq. 26). To do this, we differentiated Eq. 7, equating the result to zero by assigning *x* = *w*. As a result, we found that SM^*r*^ (Eq. 7) will comply with the Main Axiom conditions only if

40 $$r = R - \frac{{\sqrt 3 L^{2} Bw}}{{2(L^{2} - 4w^{2} )\sqrt {L^{2} - 4w^{2} } }}$$

The obtained Eq. 40 can be transformed into the following form, which is more suitable for subsequent analysis:

41 $$\frac{r}{B} = \frac{R}{B} - \frac{\sqrt 3 }{{2\left[ {1 - 4\left( \frac{w}{L} \right)^{2} } \right]\sqrt {\left( \frac{w}{L} \right)^{ - 2} - 4} }}$$

We noted above that the value of the radius *R* in the blunt part of the egg would be the same for all varieties of the pointed end shape and is determined using Eq. 20. Then, Eq. 41 will take the following form:

42 $$\frac{r}{B} = \frac{\sqrt 3 }{2}\left[ {\frac{1}{{2\sqrt {1 - 2\frac{w}{L} + 4\left( \frac{w}{L} \right)^{2} } }} - \frac{1}{{\left( {1 - 4\left( \frac{w}{L} \right)^{2} } \right)\sqrt {\left( \frac{w}{L} \right)^{ - 2} - 4} }}} \right]$$

**Fig. 5:**
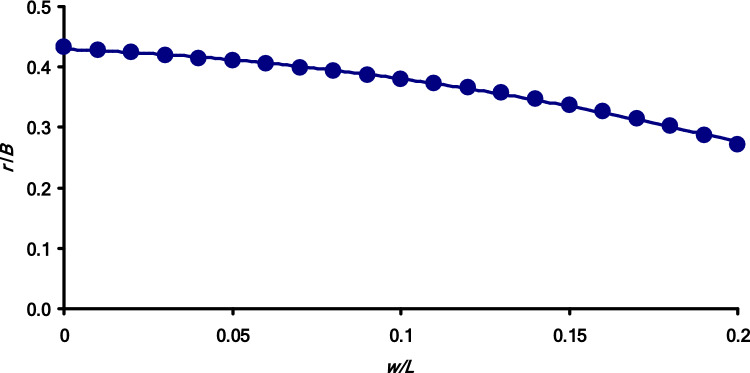
Visualized representation of Eq. 42 as a graphical dependence of the *r*/*B* ratio on the *w*/*L* ratio

Taking advantage of the fact that the range of *w*/*L* value variations for bird eggs is very limited and is [0 … 0.2] (Narushin et al. 2021, 2022, 2023a, b), we visualized Eq. 42 as the appropriate graphical dependence (Fig. 5). Although the theoretically possible variation of *w*/*L* ratio can be as high as 0.25 (Narushin et al. 2021), in later studies of actual eggs of all shapes and sizes (Narushin et al. 2022, 2023a, b), we demonstrated that the maximum *w*/*L* value was 0.14. Thus, making some allowance for the possible presence of super-elongated eggs, we set the upper value of *w*/*L* at 0.2, which should be more than sufficient to satisfy the visual representation of all virtual bird egg shapes.

Thus, the compliance of SM^*r*^ (Eq. 7) with the Main Axiom conditions is possible only for eggs with a certain ratio of parameters determined by the dependence in Fig. 5. At the same time, the variations in the *r*/*B* values are very limited and change in the range of 0.27 … 0.43. Nevertheless, we decided to conduct a practical assessment of a certain “symbiosis” of the SM^MA^ (Eq. 26) and SM^*r*^ (Eq. 7) models. Comparison of Eqs. 42 and 25 allowed us to transform Eq. 42 into the following equation:

$$r = \frac{\sqrt 3 BL}{{4\sqrt {L^{2} - 2wL + 4w^{2} } }} - \tan \theta \cdot \frac{\sqrt 3 L}{4}$$Wherefrom,

43 $$\tan \theta = \frac{B}{{\sqrt {L^{2} - 2wL + 4w^{2} } }} - \frac{4r}{{\sqrt 3 L}}$$

Substituting Eq. 43 into the original model Eq. 5, allowed us to derive another modification that we assigned the superscript index ‘U’ as the supposed ‘universal’ SM. Then, SM^U^ would be as follows:

44 $$y = \pm B\left( {\frac{1}{{2\sqrt {L^{2} - 4w^{2} } }} + \left[ {\frac{1}{{\sqrt {L^{2} - 2wL + 4w^{2} } }} - \frac{4r}{{\sqrt 3 BL}}} \right]\frac{x - w}{L}} \right)\sqrt {L^{2} - 4x^{2} }$$

**Fig. 6:**
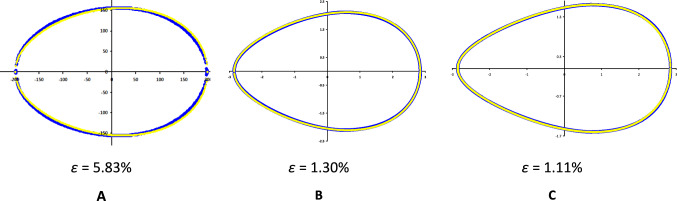
Visualization results of geometric profiles obtained using SM.^U^, Eq. 44 (yellow line), compared with actual egg contours of three bird species (blue line): chicken (*Gallus gallus*) egg (**A**); common sandpiper (*Actitis hypoleucos*) egg (**B**); razorbill (*Alca torda*) egg (**C**)

Testing the practical application of the obtained modification (Eq. 44) on the same egg profiles yielded the results shown in Fig. 6.

Unfortunately, SM^U^ (Eq. 44) did not demonstrate either a clear increase in the accuracy of geometric reproduction of egg contours or compliance with the Main Axiom conditions. The only advantage of this model can be recognized as achieving a sufficiently high degree of contour copying using four initial egg measurements (*L*, *B*, *w* and *r*).

The only modification of the SM proposed and described by Eq. 26 takes into account the requirements of the Main Axiom condition (Narushin et al. 2023a). In the same work (Narushin et al. 2023a), we demonstrated the practical benefit of having mathematical models that strictly correspond to geometric laws. Even Plato (c. 427–c. 347 BC) postulated the perfection of geometric forms, which he called *universals* (e.g., Moreland 2001). According to Plato, universals consist of ideal forms, while physical objects are just their imperfect copies. Thus, based on the principles of this doctrine of forms, the presence of an “ideal” geometric model of the egg as expressed by Eq. 26 allows us to have a certain standard to which the profile of an actual bird’s egg approaches. By assessing the degree of difference between the actual and ideal shape, one can judge how much a given egg deviates from its geometric standard. In this regard, the mathematical modification we called SM^MA^ (Eq. 26), since it is the only one of the derived formulas that satisfies the Main Axiom principles, can be a good alternative to the so-called “universal” egg formula we derived earlier (Narushin et al. 2023a).

The issues of certain fundamental principles adopted by Smart (1969) as the basis for creating his model, as demonstrated by us here in the course of theoretical studies, do not diminish its significance as the first and, to date, the only mathematical formula based on the physiological principles of egg formation. The process of giving the egg an ovoid shape due to mechanical compression of the oviduct walls of the mother’s body remains outside the assumptions of Smart (1969), according to which the initially spherical egg is transformed into an ellipsoid, after which it is compressed at a certain point, although at different compression angles to the shape of an ovoid. Hereby, the length formed at the ellipsoid shape stage remains unchanged. Such a scenario is logical and quite simple from the view point of performing this work by some mechanical device. Biological systems operate in a much more sophisticated and intricate manner, however, breaking our stereotypes about how to make this process easier, working on different principles and adhering to different, obviously more stringent and vital standards.

Therefore, the question of the relationship between the physiological process of egg formation and its mathematical model remains open. In a very recent study, Deeming (2024) described the physiological principles of egg formation in the bird’s oviduct, laying the foundation for a possible description of this mechanism in the language of mathematical symbols. Thus, the “symbiosis” of biological and mathematical premises can result in a new model by revising the principles of geometric standards and universal approaches to the description of all the diversity of bird egg shapes existing in nature.

## Conclusions

The dilemma in implementing the SM for practical purposes is that a sufficiently high reproduction accuracy of the graphic model of the bird’s egg contours is possible when using measurements of at least four initial parameters: *L*, *B*, *w* and *r*. In this case, the mathematical model expressed by Eq. 44 should be used. Instead of the *r* value, it is permissible to use the measurement of *B*_0_ and the appropriate mathematical model expressed by Eq. 9. Both equations give comparable results of accuracy that will be sufficient for practical implementation. The usage of five measurements, where, in addition to the specified *L*, *B*, *w* and, *R* is also applied, provides the highest accuracy of geometric reproduction when using the mathematical model expressed by Eq. 7. However, these modifications of SM violate the principles of geometric laws, changing the coordinates of the extremum point. The only one of the proposed SM modifications that is described by Eq. 26 takes into account the requirements of this condition, which we called the “Main Axiom of the Mathematical Formula of the Bird’s Egg”. Its use does not demonstrate the absolute accuracy of geometric reproduction of the actual egg contour. At the same time, it has the undoubted advantage of claiming a standard geometric figure that can be conventionally called an “*egg-shaped profile*”, which means eggs of different types, from spherical to ellipsoid, to pyriform.

Two postulates taken by Smart (1969) as the basis for the mathematical description of the physiological process of egg formation, i.e., the application point of the oviduct force to give the appropriate egg shape, and the equality of *L* and *L*_*el*_, turned out to be erroneous. This was demonstrated by the theoretical studies presented here. Nevertheless, Smart’s (1969) attempt to derive a formula based on the physiology of egg formation is a pioneering and promising approach to the development of appropriate mathematical models for describing the geometric features of the bird egg shape.

## Data Availability

All data supporting the findings of this study are available within the paper and its supplementary data.
